# Exploring glucocorticoid dose–response patterns in VEXAS syndrome: a pilot retrospective study

**DOI:** 10.1007/s00296-026-06130-3

**Published:** 2026-05-19

**Authors:** Nicolas Giachetti, Jérome Hadjadj, Noémie Gaudré, Christian Lavigne, Valentin Lacombe

**Affiliations:** 1 Médecine interne - Immunologie clinique, Centre National de Référence Maladies Auto-immunes Systémiques Rares du Nord, Nord-Ouest, Méditerranée et Guadeloupe (CeRAINOM-Angers), CHU d’Angers, Angers, France; 2 Service de medecine interne, Sorbonne Université, Hôpital Saint-Antoine, CEREMAIAA, AP-HP, Paris, France; 3 Center for Human Genetics and Genomics, New York University Grossman School of Medicine, New York, NY USA; 4 Médecine interne et polyvalente, CH du Haut-Anjou, 1 quai du Dr Lefèvre, 53200 Château-Gontier, France; 5 Univ Angers, Inserm, CNRS, MITOVASC, Equipe MitoLab, SFR ICAT, 49000 Angers, France

**Keywords:** VEXAS syndrome, *UBA1* mutation, Glucocorticoid tapering, Glucocorticoid dependence, Disease flare, Steroid-sparing therapies

## Abstract

VEXAS syndrome is characterized by high glucocorticoid (GC) requirements and frequent relapses during tapering, yet dose–response relationships remain poorly defined. We conducted a pilot study to assess the feasibility of granular longitudinal treatment-line reconstruction and to explore GC dose–response patterns associated with disease activity and flare risk. In this retrospective single-center study, we reconstructed detailed treatment trajectories of patients with VEXAS syndrome. Disease courses were segmented into treatment-line periods defined by any therapeutic modification, including GC dose changes. For each period, GC dose, concomitant therapies, and clinical activity were recorded. Associations between GC dose and disease activity were analyzed using multivariable models adjusting for concomitant treatments. Twelve patients were included, representing 315 treatment-line periods over a mean follow-up of 45 months. Mean annual GC exposure was 3.27 ± 2.26 g per patient. Higher GC dose (continuous variable) was independently associated with lower odds of clinical activity (aOR 0.91 per mg increase [95% CI 0.88–0.94], p < 0.001). Exploratory threshold analyses suggested increased flare risk below approximately 15 mg/day (or 0.2 mg/kg/day), with a lower-risk plateau above 20 mg/day. The increased flare risk below 15 mg/day remained significant after adjustment for concomitant therapies (HR 0.24 [95% CI 0.11–0.54], p = 0.001). Organ-specific analyses suggested heterogeneous glucocorticoid sensitivity, with higher doses appearing required for lung involvement and lower doses appearing sufficient for fever, skin, and joint manifestations. This pilot study demonstrates the feasibility of detailed treatment-line reconstruction in VEXAS syndrome and identifies GC dose–response trends. These preliminary findings provide planning parameters for a future adequately powered study aimed at validating dose thresholds and refining tapering strategies in VEXAS.

**Supplementary Information:**

The online version contains supplementary material available at 10.1007/s00296-026-06130-3.

## Introduction

VEXAS (Vacuoles, E1 enzyme, X-linked, Autoinflammatory, Somatic) syndrome is a late-onset autoinflammatory disorder caused by somatic *UBA1* mutations in hematopoietic stem cells [1]. It predominantly affects older men and combines systemic inflammatory manifestations [2–5] with hematological abnormalities [2, 6]. Despite increasing recognition of the disease, therapeutic management remains challenging. In most cases, treatment relies on IL-6 inhibitors, JAK inhibitors, and azacytidine [7–11], although anti-IL6 and JAK inhibitors did not demonstrate a clear glucocorticoid-sparing effect in a recent prospective study [12].

Recent data and expert guidance highlight the substantial glucocorticoid (GC) dependence of VEXAS, with frequent relapses during tapering making GC reduction a major therapeutic challenge [13–15]. Indeed, long-term GC use is associated with significant toxicity [16], which may be magnified in this older and immunocompromised population [17–19]. Conversely, overly aggressive tapering may precipitate flares and hospitalizations. Yet, GC dose–response relationships in VEXAS remain poorly characterized. In particular, the levels at which disease control is maintained, the dose ranges associated with increased flare risk, and potential variability across organ manifestations have not been systematically explored.

Because VEXAS is a recently described and heterogeneous disease, large registries may lack sufficient granularity to reconstruct detailed treatment trajectories. We therefore conducted a pilot retrospective study aimed at assessing the feasibility of granular longitudinal treatment-line reconstruction and exploring GC dose–response patterns associated with disease activity. This exploratory analysis was designed to generate hypotheses and to provide parameters for a future adequately powered study aimed at validating dose thresholds and refining tapering strategies in VEXAS.

## Methods

### Ethics

This study was approved by the Angers University Hospital Ethics Committee (Comité d’Ethique du Centre Hospitalier Universitaire d’Angers, approval number 2022-055, on April 5, 2022), conducted in accordance with the Declaration of Helsinki, and reported following the STROBE (Strengthening the Reporting of Observational Studies in Epidemiology) guidelines, in line with EQUATOR Network recommendations (Supplementary Data S1). Written informed consent for publication was obtained from all living patients.

### Study design and population

We conducted a retrospective single-center pilot study at Angers University Hospital (France). All patients with genetically confirmed VEXAS syndrome followed between January 2010 and December 2023 were included. For each patient, disease history was retrospectively reconstructed from the first symptoms attributable to VEXAS until death, loss to follow-up, or the end of the study period. Given the recent recognition of VEXAS [1] and the even more recent publication of management recommendations [15], early treatment strategies were not standardized.

### Treatment-line reconstruction

For each patient, a detailed retrospective chart review was performed. The disease course was reconstructed from the first manifestation retrospectively attributed to VEXAS.

Treatment history was segmented into treatment-line periods, defined by any therapeutic modification (initiation, discontinuation, substitution, or dose adjustment), including GC tapering or escalation. For each period, we recorded GC dose (mg/day and mg/kg/day prednisone-equivalent), patient’s weight, concomitant therapies, duration, clinical activity status, and clinical manifestations.

### Definition of disease activity

Disease activity was clinically defined according to the international consensus definition of VEXAS flare [20]. Each treatment-line period was classified as “Inactive” (absence of inflammatory manifestations, regardless of GC dose) or “Active”, subdivided into “Flare” (new or recurrent inflammatory manifestations) and “Persistent activity” (ongoing symptoms without an intervening inactive period).

CRP was not included in activity definition due to limited reliability under IL-6 blockade and infection risk in this population.

### Statistical analysis

Given the exploratory nature of this pilot study and the limited number of patients, analyses were primarily descriptive and hypothesis-generating.

We performed two complementary exploratory analyses: i) Activity analysis, assessing associations between GC dose and active versus inactive treatment-line periods, and ii) Flare analysis, evaluating GC dose in relation to relapse-free survival.

Associations between GC dose and disease activity were explored using logistic regression models adjusted for concomitant therapies. Time-to-flare was analyzed using Kaplan–Meier curves and Cox proportional hazards models. GC dose was analyzed as a continuous variable and across predefined categories.

As a sensitivity analysis, the main model assessing the association between GC dose and disease activity was repeated using a mixed-effect logistic regression, including patient ID as a random intercept, to account for intra-patient clustering, and adjusting for concomitant therapies.

Concomitant therapies were included as adjustment variables in the models. For the activity analysis, each concomitant treatment was entered individually as a categorical variable, whereas for survival analyses, therapies were grouped into three categories: targeted agents with previously reported higher efficacy in VEXAS (ruxolitinib, tocilizumab, or azacytidine [15]), other immunosuppressive therapies, and GC alone.

Threshold analyses using maximally selected rank statistics were performed for exploratory identification of potential dose ranges associated with flare risk.

Quantitative data are presented as mean ± SD and categorical data as counts (%). A two-sided alpha of 0.05 was used. Analyses were performed using GraphPad Prism and Jamovi.

Detailed statistical methods are provided in the *Supplementary Methods*.

### Search strategy for the narrative review in discussion

A targeted literature search was conducted in accordance with the CABARET recommendations of the EQUATOR Network to contextualize the findings of this study [21]. The Medline/PubMed database was searched for relevant publications up to December 2025. The search strategy combined terms related to VEXAS syndrome (“VEXAS”, “UBA1”) and its therapeutic management (“glucocorticoids”, “corticosteroids”, “treatment”, “therapy”). We included observational cohorts, clinical trials, and review articles reporting on glucocorticoid exposure, disease activity, or steroid-sparing therapies in VEXAS syndrome. Additional key references were identified through manual screening of reference lists and relevant guidelines. To further contextualize our findings, we also searched for studies addressing glucocorticoid tapering strategies in related inflammatory and autoimmune diseases (e.g., giant cell arteritis, systemic lupus erythematosus, and ANCA-associated vasculitis), restricting inclusion to clinical trials. Finally, we examined the literature on glucocorticoid mechanisms of action, including both fundamental research studies and review articles, to support the biological interpretation of our results.

## Results

### Study population

Twelve male patients were included (mean age at onset 69.1 ± 8.7 years), with a mean follow-up of 45 ± 32 months. Clinical and biological characteristics are summarized in Table 1. All patients received glucocorticoids during their disease course. Concomitant therapies were heterogeneous, reflecting evolving management strategies over time (Table 1).

**Table 1:** Characteristics of the study population

	Study population (n = 12)
General characteristics	
Male (n, %)	12 (100%)
Age at symptom onset (years, mean ± SD)	69.1 ± 8.7
Age at diagnosis (years mean ± SD)	73.3 ± 8.9
Duration of follow-up (months, mean ± SD)	45.0 ± 31.8
*UBA1* mutation (n, %)	
p.Met41Thr	4 (33.0%)
p.Met41Leu	3 (25.0%)
p.Met41Val	4 (33.3%)
Splice	1 (8.3%)
Clinical features during the disease course *(n, %)*	
Fever	11 (91.7%)
Night sweats or impaired general condition	5 (41.7%)
Joint involvement	8 (66.7%)
Chondritis	7 (58.3%)
Skin involvement	12 (100%)
Lung involvement	9 (75.0%)
Ocular or periorbital involvement	8 (66,7%)
Biological features during the disease course (n, %)	
CRP elevation (> 10 mg/l)	12 (100%)
Anemia (< 13 g/dl in men)	10 (83.3%)
Macrocytosis (> 98 fl)	9 (75.0%)
Lymphopenia (< 1 G/L)	11 (92.7%)
Severe lymphopenia (< 0.5 G/L)	8 (66.7%)
Thrombocytopenia (< 150 G/L)	8 (66.7%)
Severe thrombocytopenia (< 50 G/L)	3 (25.0%)
Treatment received during the disease course (n, %)	
Glucocorticoids	12 (100%)
NSAIDs	1 (8.3%)
Methotrexate	2 (16.7%)
Cyclophosphamide	1 (8.3%)
Anti-TNF⍺	2 (16.7%)
Anti-IL1	3 (25.0%)
Anti-IL6R	4 (33.3%)
Ruxolitinib	4 (33.3%)
Tofacitinib	1 (8.3%)
Azacytidine	1 (8.3%)

Values are expressed as mean (standard deviation) for continuous variables and absolute values (percentages) for categorical variables

*Anti-IL1* anti-interleukin-1 (anakinra or canakinumab), *Anti-IL6R* anti-interleukin-6 receptor (tocilizumab), *Anti-TNF⍺ *anti-tumor necrosis factor alpha (infliximab or etanercept), *CRP* C-reactive protein, *NSAIDs* non-steroidal anti-inflammatory drugs

Across the cohort, 315 treatment-line periods were reconstructed (mean duration 37 ± 94 days), of which 88 (28%) were classified as active, including 41 flare periods and 47 periods of persistent activity.

### Glucocorticoid exposure

GC therapy accounted for a mean of 54.1% ± 31.2 of the total follow-up time. During the first year of treatment, the cumulative GC exposure reached 5 369 ± 2 428 mg. Over a mean follow-up of 3.7 ± 2.6 years, total cumulative dose was 9 885 ± 6 827 mg per patient, (annual exposure of 3 266 ± 2 257 mg).

**Fig. 1:**
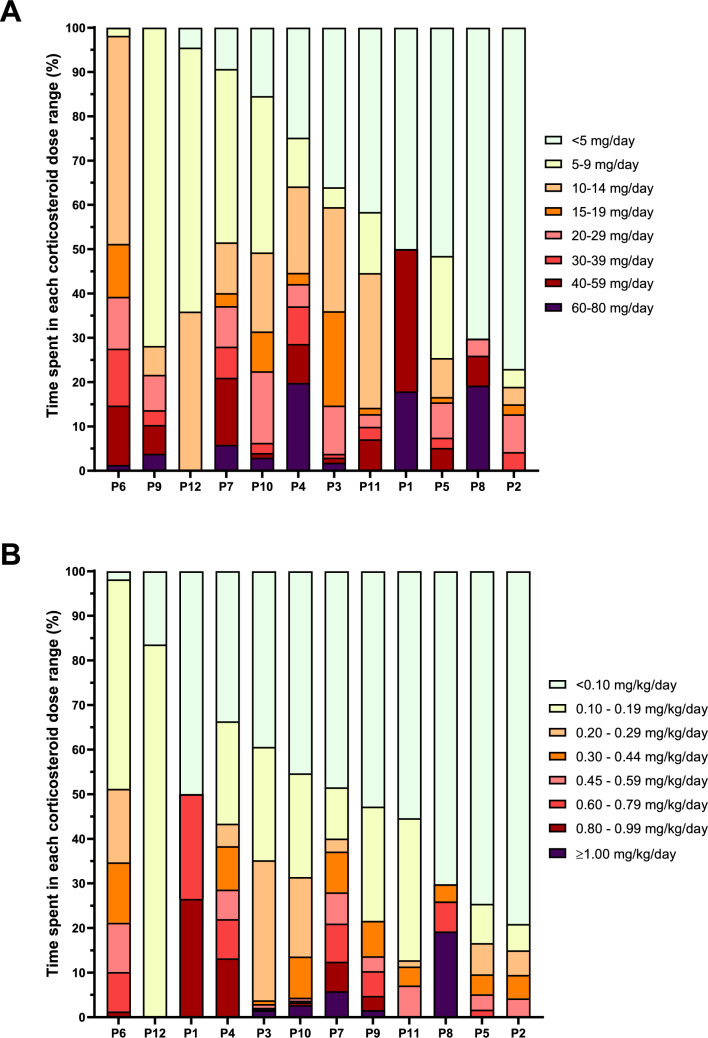
Time distribution across glucocorticoid dose categories during follow-up. Each bar represents a single patient, showing the percentage of follow-up time spent within predefined glucocorticoid dose categories, expressed as either absolute doses in mg/day (**A**) or weight-adjusted doses in mg/kg/day (**B**)

Half of the patients (6/12) were exposed to > 10 mg/day for more than half of the follow-up, and half (6/12) to > 40 mg/day for over 10% of the follow-up (Fig. 1). Individual GC trajectories, including concomitant treatments and activity status, are shown in Supplementary Data S2.

When stratified by *UBA1* variant, patients with the p.M41V mutation showed a trend toward higher mean daily GC requirements compared with p.M41L and p.M41T (21.1 ± 7.4 vs. 10.5 ± 3.2 and 13.2 ± 5.6 mg/day), although this difference did not reach statistical significance (p = 0.13, ANOVA; Supplementary Data S3).

GC-related complications included severe infections (5/12, including one fatal case), diabetes (3/12), osteoporotic fractures (2/12), and psychiatric decompensation (1/12); 6/12 experienced > 10% weight gain during follow-up.

### Glucocorticoid dose and disease activity

The proportion of time spent inactive was higher under GC ≥ 20 mg/day compared with < 20 mg/day (95.4% vs 48.6%, p < 0.0001).

In multivariable logistic regression adjusting for concomitant therapies (included individually in the model), higher GC dose (continuous variable) was independently associated with lower odds of disease activity (aOR 0.91 [95%CI: 0.88–0.94] per 1 mg increase, p < 0.001). Results were consistent in sensitivity analyses using mixed-effects logistic regression accounting for intra-patient clustering and adjusting for concomitant therapies (OR = 0.92 [95%CI: 0.88—0.96], p < 0.001). When categorized, doses > 15 mg/day were independently associated with inactive disease (Supplementary Data S4).

In exploratory organ-specific analyses, lung involvement appeared less frequently active at higher GC doses (> 15—20 mg/day), whereas skin, fever, and joint manifestations were less frequent even at moderate doses (5—15 mg/day); no association was observed for chondritis, ocular involvement, or constitutional symptoms (Supplementary Data S5).

### Risk of flare according to the glucocorticoid dose

**Fig. 2:**
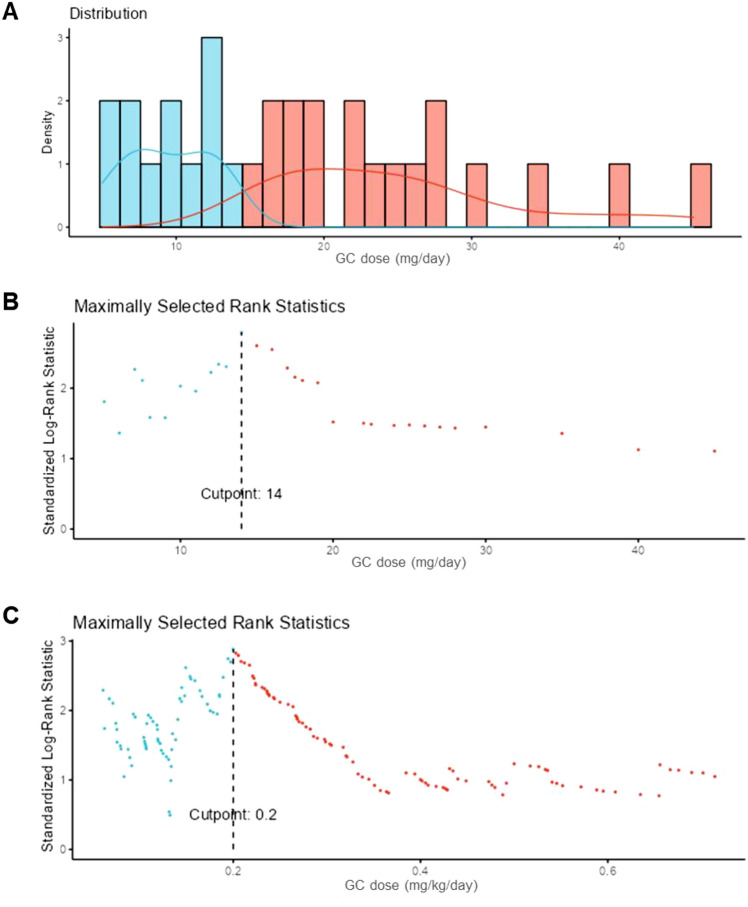
Identification of glucocorticoid dose thresholds associated with flare risk in VEXAS syndrome. **A** Distribution of prednisone-equivalent daily doses (mg/day) across treatment-line periods. Bars represent the relative frequency (density) of observations at each dose level, allowing comparison of how often different dose ranges occur in the dataset. Overlaid curves show smoothed density estimates, which provide a continuous approximation of the dose distribution within each group (blue: below threshold; red: above threshold), facilitating visual comparison of their overall patterns. **B** Maximally selected rank statistics for absolute GC dose (mg/day). Each point corresponds to a candidate threshold and represents the standardized log-rank statistic measuring the difference in flare-free survival between groups defined by that threshold. Higher values indicate greater discrimination between groups. The dashed vertical line marks the dose (14 mg/day) at which this separation is maximal in this dataset. **C** Maximally selected rank statistics for weight-adjusted GC dose (mg/kg/day), following the same approach as in panel B. The dashed vertical line (0.2 mg/kg/day) indicates the dose associated with the greatest separation between groups. *GC* glucocorticoids

In exploratory threshold analyses, dose ranges appeared to be associated with differential flare risk. A lower-risk plateau was observed above ~ 0.35 mg/kg/day (or ~ 20 mg/day), whereas increased flare risk appeared to be observed below ~ 0.2 mg/kg/day (or ~ 15 mg/day) (Fig. 2).

**Fig. 3:**
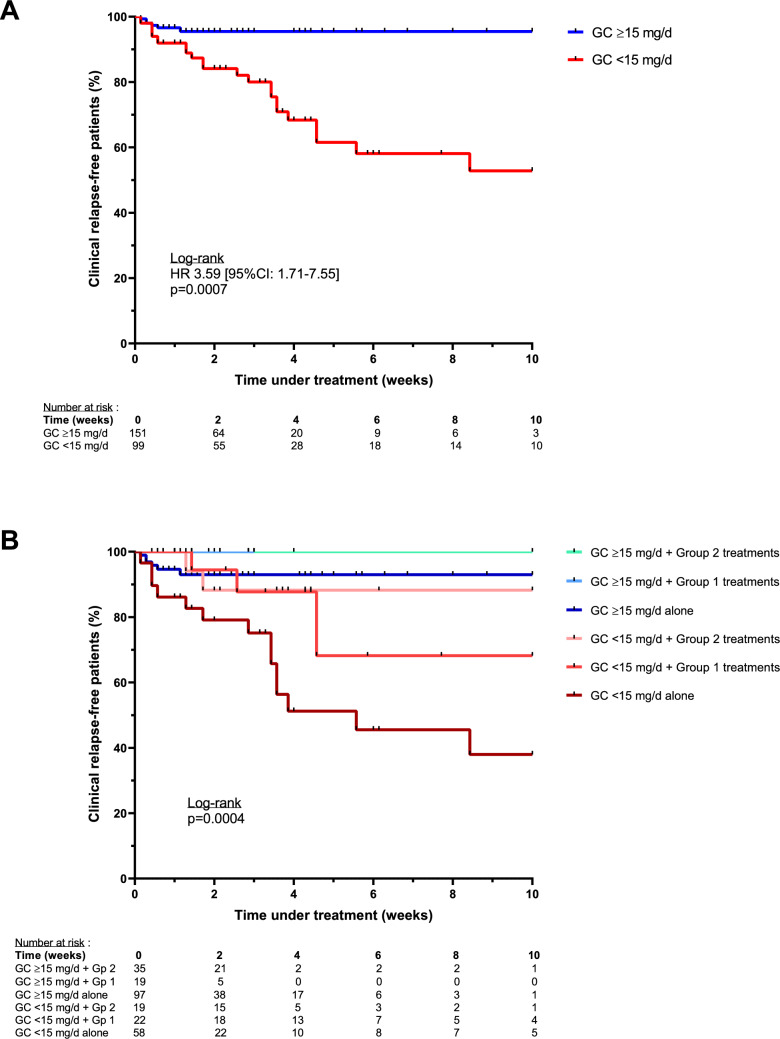
Relapse-free survival according to glucocorticoid dose and concomitant treatment. **A** Kaplan–Meier curve comparing relapse-free survival under < 15 mg/day vs ≥ 15 mg/day prednisone-equivalent doses, regardless of concomitant therapy. **B** Stratified analysis showing relapse-free survival by glucocorticoid dose and treatment group: Group 2 (ruxolitinib, tocilizumab, or azacitidine), Group 1 (other immunosuppressive agents), or GC alone. GC: glucocorticoids

In univariate Kaplan–Meier analysis, prednisone < 15 mg/day was associated with higher flare risk (HR 3.59 [95%CI: 1.71—7.55], p = 0.0007; Fig. 3A). In multivariable Cox regression adjusting for concomitant therapies, GC dose remained associated with flare risk both as a continuous variable (HR 0.95 [95%CI: 0.92—0.98] per mg/day, p = 0.004) and when dichotomized at 15 mg/day (HR 0.24 [95%CI: 0.11—0.54], p = 0.001). Similar associations were observed using the 0.2 mg/kg/day threshold.

When stratified by concomitant therapy, relapse-free survival was explored across GC dose categories (< 15 vs ≥ 15 mg/day) and three treatment groups: targeted agents with previously reported higher efficacy in VEXAS (ruxolitinib, tocilizumab, or azacytidine [15]), other immunosuppressive therapies, and GC alone. Under ≥ 15 mg/day, relapse-free survival appeared comparable across treatment groups. In contrast, under < 15 mg/day, flare risk varied according to concomitant therapy, appearing lower with higher-efficacy agents and higher with other immunosuppressive therapies or GC alone (p = 0.0004, log-rank test; Fig. 3B).

## Discussion

Recent publications have substantially improved the characterization of VEXAS syndrome. Large-scale studies have detailed the clinical spectrum, genetic features, and overall management patterns across several hundred patients [2, 4, 11]. More focused reports have highlighted the heterogeneity of clinical presentations and therapeutic responses, as well as the challenges of diagnosis and treatment selection [7, 9, 22]. However, most studies have primarily relied on cohort-level analyses and do not capture the detailed, individual longitudinal trajectories of treatment response. In this context, our study offers a complementary perspective by reconstructing individual treatment trajectories at a granular level and quantitatively exploring glucocorticoid dose–response relationships in relation to both disease activity and flare risk. This approach enables a more precise assessment of treatment patterns over time and may help inform more structured tapering strategies in clinical practice.

To date, GC tapering and dose adjustment in VEXAS syndrome remain empirical [15], extrapolated from practices in other inflammatory and autoimmune diseases [23, 24]. Previous studies and recent ACR guidance have highlighted the high GC requirements in VEXAS syndrome, the difficulty of tapering without triggering flares, and the rarity of patients achieving GC-free remission even with GC-sparing agents [2, 7, 10, 15, 25]. In this pilot study of 12 patients, the granular reconstruction of 315 treatment-line periods demonstrated the feasibility of detailed retrospective longitudinal dose tracking, enabling exploratory dose–response analyses linking GC exposure to both time-to-flare and overall disease activity. Across analytical approaches, higher doses were associated with lower odds of activity and reduced flare risk, with exploratory analyses suggesting increased relapse below approximately 15 mg/day and low flare risk above 20 mg/day in this cohort. Differences according to concomitant therapies were only observed at lower doses in this small sample; confirmation in a larger, adequately powered study will be required. Finally, based on these preliminary data, some clinical manifestations seemed more sensitive to glucocorticoids than others.

Compared to other inflammatory diseases, our findings suggest that VEXAS may be particularly GC-dependent. In this cohort, relapse risk seemed to increase below 15–20 mg/day, whereas lower relapse thresholds are reported in giant cell arteritis (≈10 mg/day) [26] and systemic lupus erythematosus (≈5 mg/day) [27]. Similarly, VEXAS-related polychondritis has been associated with more refractory disease and higher GC requirements than idiopathic relapsing polychondritis [25]. This apparent GC dependency translated into substantial cumulative exposure, with a mean annual dose of 3,266 mg per patient and a first-year exposure of 5,369 ± 2,428 mg—numerically higher than those reported in prospective trials of giant cell arteritis (3,818 mg) [23] and ANCA-associated vasculitis (3,847 mg). In line with this, our cohort exhibited several complications potentially related to glucocorticoid exposure, including severe infections, metabolic disorders, and osteoporotic events. However, many patients in our cohort experienced prolonged diagnostic delay and were treated before VEXAS was recognized and before current therapeutic recommendations were available, which may have contributed to higher cumulative GC exposure. The p.M41V variant, previously linked to more severe disease [28], may also be associated with greater corticosteroid requirements, although this observation remains exploratory. Collectively, these findings reinforce the need for effective steroid-sparing strategies in VEXAS, given the substantial cumulative GC exposure observed and the heightened susceptibility of these patients to dose-dependent infectious and metabolic complications.

These findings highlight the urgent need for steroid-sparing strategies in VEXAS, given the well-established risks of prolonged high-dose GC therapy [15]. In other autoimmune and inflammatory conditions, high cumulative or long-term GC exposure has been associated with serious adverse outcomes, including infections, osteoporosis, diabetes, and increased mortality [29–34]. This is particularly concerning in VEXAS syndrome, where intrinsic immune dysfunction (monocytopenia, lymphopenia, impaired NK-cell and monocyte function) [35–37] already predisposes patients to severe and opportunistic infections [17–19]. Importantly, the risk of GC-related infections is strongly dose dependent, rising above 10 mg/day or with cumulative doses exceeding 700 mg [38], thresholds that are frequently surpassed in VEXAS patients.

Our findings are broadly consistent with recent data from Turturice et al*.*, who reported persistent GC dependence in VEXAS, with mean prednisone doses remaining above 15 mg/day and rare remission under ≤ 10 mg/day despite IL-6 or JAK inhibition [12]. However, whereas cytokine-directed therapies did not demonstrate a clear steroid-sparing effect in their study, our small cohort suggested a modest reduction in relapse risk with agents previously reported as higher efficacy (ruxolitinib, tocilizumab, azacitidine), but only in case of GC doses < 15 mg/day, although this signal must be interpreted cautiously given our limited sample size. Because azacitidine was not assessed in their analysis and was administered to only one patient in ours, its effect could not be evaluated here; however, its steroid-sparing potential has been supported by Jachiet et al*.* [9]. Together with prior reports [7, 9], these preliminary observations support the need for prospective studies in VEXAS that explicitly incorporate glucocorticoid exposure and steroid-sparing efficacy as key therapeutic outcomes.

Beyond its exploratory findings, this study especially provides key parameters for the design of a future adequately powered multicenter investigation: the observed event accrual, variability in GC exposure, and effect-size estimates for both activity and flare endpoints offer realistic planning inputs for confirmatory analyses. A larger collaborative study will be required to validate dose–response relationships and phenotype-informed tapering strategies in VEXAS.

The mechanisms underlying the marked corticosteroid dependency in VEXAS syndrome remain unclear, but several hypotheses emerge from our findings and the known pathophysiology. VEXAS results from somatic mutations in *UBA1*, encoding the E1 enzyme that initiates ubiquitination [1]—a key regulator of glucocorticoid receptor (GR) degradation, trafficking, and transcriptional activity [39, 40]. Impaired ubiquitination may disrupt GR signaling and alter corticosteroid responsiveness. Although ubiquitination usually inhibits GC signaling, its defect could interfere with the resolution or modulation of GC effects. The predominance of clinical responses at high GC doses suggests that non-genomic pathways—engaged at higher doses—may remain functional, whereas genomic responses could be either impaired in VEXAS or effective only for certain manifestations [41, 42]. Non-genomic GC effects, such as mitochondrial destabilization and activation of apoptotic pathways [41, 43], might directly affect clonal cells or indirectly reduce clonal selection by modulating the inflammatory bone marrow niche [44]. By contrast, genomic effects, typically elicited at lower doses, may explain why some symptoms driven by paraclonal inflammation (fever, rash, arthritis) improve with moderate GC exposure [45]. Mechanistic studies are needed to investigate how GC act on both *UBA1*-mutated and non-mutated cells in VEXAS, as both contribute to disease-related inflammation [35], but may differ in their GC responsiveness.

Despite the detailed reconstruction of 315 treatment-line periods, the small number of patients limits statistical power, restricts the assessment of inter-individual heterogeneity and treatment interactions, and reduces the generalizability of organ-specific findings. Multiple treatment-line periods introduce non-independence and a risk of pseudo-replication, partly addressed by mixed-effects models, although survival analyses were not adjusted for intra-patient clustering. Although treatment-line periods incorporated timing and duration of exposure, cumulative exposure over time was not explicitly modeled. The retrospective design may have led to incomplete data capture, unmeasured treatment non-adherence, and underestimation of corticosteroid-related complications. The clinical definition of disease activity allowed focus on clinically relevant flares but may introduce subjectivity. Therapeutic exposures were heterogeneous, partly reflecting prolonged diagnostic delay and management prior to recognition of VEXAS and the publication of treatment recommendations, which may have influenced cumulative GC exposure. Also, confounding by indication cannot be excluded, as higher glucocorticoid doses may have been used in more severe disease, potentially biasing the observed associations. As a pilot study, the aim was not to provide definitive thresholds but to explore feasibility, identify trends, and generate preliminary parameters to inform the design of a larger, adequately powered study. Therefore, the identified dose thresholds should be considered exploratory and require validation in larger cohorts.

## Conclusion

In this pilot study, we demonstrate the feasibility of detailed longitudinal reconstruction of therapeutic trajectories in VEXAS, enabling fine-grained analysis of glucocorticoid exposure in relation to disease activity and flare risk. In this small cohort, exploratory analyses suggested increased relapse risk below 15 mg/day, relatively low flare risk above 20 mg/day, and potential variation in glucocorticoid sensitivity across clinical manifestations. Differences according to concomitant therapies were mainly observed at lower doses, whereas at higher doses such effects were not clearly detectable in this limited sample, underscoring the importance of explicitly integrating glucocorticoid dose into response definitions and outcome measures in future prospective studies. These findings should be considered hypothesis-generating rather than definitive. The primary contribution of this work is to delineate preliminary dose–response signals and provide quantitative and methodological groundwork for a larger, prospective, adequately powered study. Such studies will be required to validate dose thresholds and phenotype-informed tapering strategies in VEXAS.

## Supplementary Information

Below is the link to the electronic supplementary material.

Supplementary file1 (PDF 187 KB)

Supplementary file2 (PDF 107 KB) Supplementary Data S2. Evolution of glucocorticoid dosing over the disease course, concomitant therapies and disease activity status. Notes: Glucocorticoid doses (y-axis, prednisone-equivalent) are plotted over time (x-axis). Each line segment represents a treatment-line period, defined by a stable glucocorticoid dose and unchanged concomitant therapies, with each point indicating a change in treatment (e.g., initiation, discontinuation, or dose adjustment). Grey shaded areas indicate periods of active disease. Horizontal arrows denote the timing of concomitant therapies when applicable. This figure illustrates the individual treatment trajectories and highlights the interplay between glucocorticoid tapering, adjunctive treatments, and disease control

Supplementary file3 (PDF 5 KB) Supplementary Data S3. Corticosteroid exposure according to UBA1 variant

Supplementary file4 (PDF 191 KB) Supplementary Data S4. Multivariable logistic regression assessing the association between glucocorticoid dose and overall disease activity. Notes: Results are presented as adjusted odds ratios (aORs) with 95% confidence intervals. The model included glucocorticoid dose categories and concomitant therapies as independent variables. Treatments were analyzed individually: azacitidine, ruxolitinib, tocilizumab, anakinra or canakinumab, infliximab or etanercept, tofacitinib, methotrexate, cyclophosphamide, other, or none. Reference category was >40 mg/day for GC dose. GC: glucocorticoids

Supplementary file5 (PDF 206 KB) Supplementary Data S5. Multivariable logistic regression assessing the association between glucocorticoid dose and disease activity by clinical manifestation. Notes: Each column corresponds to a separate logistic regression model for a specific symptom. All models included glucocorticoid dose categories and concomitant therapies as independent variables. Treatments were analyzed individually: azacitidine, ruxolitinib, tocilizumab, anakinra or canakinumab, infliximab or etanercept, tofacitinib, methotrexate, cyclophosphamide, other, or none. Reference categories were <5 mg/day for glucocorticoid dose and no concomitant treatment. Constitutional symptoms include night sweats and other nonspecific systemic signs (e.g., fatigue, weight loss). GC: Glucocorticoids

Supplementary file6 (PDF 181 KB)

## Data Availability

The datasets used and analyzed during the current study are available from the corresponding author on reasonable request.

## Declarations

### Use of AI tools

The authors used ChatGPT (OpenAI, GPT-5) to improve the English language quality of the manuscript (grammar, vocabulary, and stylistic clarity). The tool was not used for study design, data collection, data analysis, or result interpretation. All AI-assisted outputs were critically reviewed, revised, edited and validated by the authors, who take full responsibility for the final content of the manuscript.
