# Supplementary material for: Exploring glucocorticoid dose–response patterns in VEXAS syndrome: a pilot retrospective study
Source: Rheumatol Int. 2026 May 19;46(6):88. doi: 10.1007/s00296-026-06130-3 (PMC13186862; doi:10.1007/s00296-026-06130-3)
Supplement: Supplementary file 2 — Supplementary file2 (PDF 107 KB) Supplementary Data S2. Evolution of glucocorticoid dosing over the disease course, concomitant therapies and disease activity status. Notes: Glucocorticoid doses (y-axis, prednisone-equivalent) are plotted over time (x-axis). Each line segment represents a treatment-line period, defined by a stable glucocorticoid dose and unchanged concomitant therapies, with each point indicating a change in treatment (e.g., initiation, discontinuation, or dose adjustment). Grey shaded areas indicate periods of active disease. Horizontal arrows denote the timing of concomitant therapies when applicable. This figure illustrates the individual treatment trajectories and highlights the interplay between glucocorticoid tapering, adjunctive treatments, and disease control [file 296_2026_6130_MOESM2_ESM.pdf]

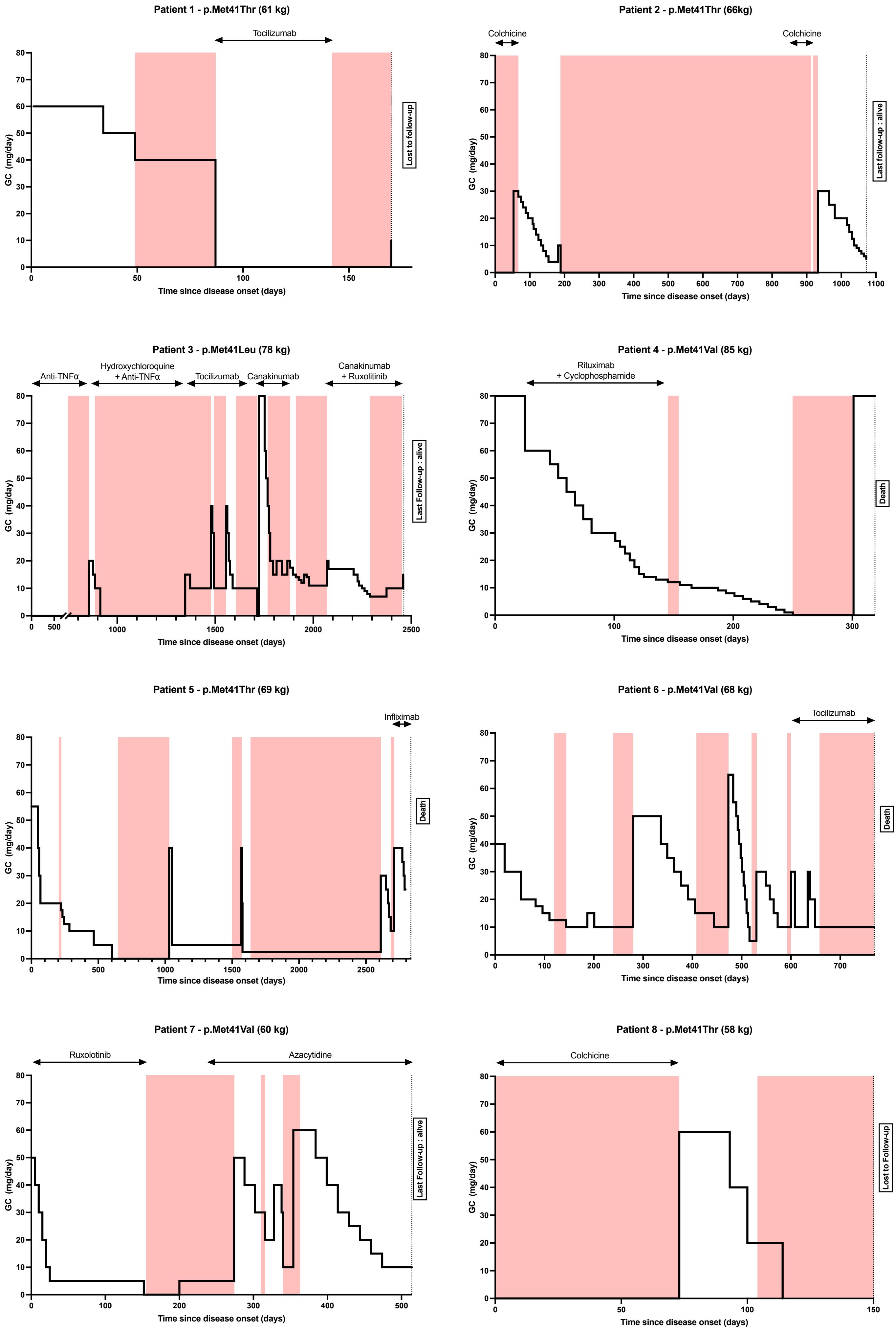

Patient 1 - p.Met41Thr (61 kg)
Tocilizumab
GC (mg/day)
Lost to follow-up
Time since disease onset (days)
Patient 2 - p.Met41Thr (66kg)
Colchicine
Colchicine
GC (mg/day)
Last follow-up : alive
Time since disease onset (days)
Patient 3 - p.Met41Leu (78 kg)
Anti-TNFα
Hydroxychloroquine
+ Anti-TNFα
Tocilizumab
Canakinumab
Canakinumab
+ Ruxolitinib
GC (mg/day)
Last Follow-up : alive
Time since disease onset (days)
Patient 4 - p.Met41Val (85 kg)
Rituximab
+ Cyclophosphamide
GC (mg/day)
Death
Time since disease onset (days)
Patient 5 - p.Met41Thr (69 kg)
Infliximab
GC (mg/day)
Death
Time since disease onset (days)
Patient 6 - p.Met41Val (68 kg)
Tocilizumab
GC (mg/day)
Death
Time since disease onset (days)
Patient 7 - p.Met41Val (60 kg)
Ruxolotinib
Azacytidine
GC (mg/day)
Last Follow-up : alive
Time since disease onset (days)
Patient 8 - p.Met41Thr (58 kg)
Colchicine
GC (mg/day)
Lost to Follow-up
Time since disease onset (days)

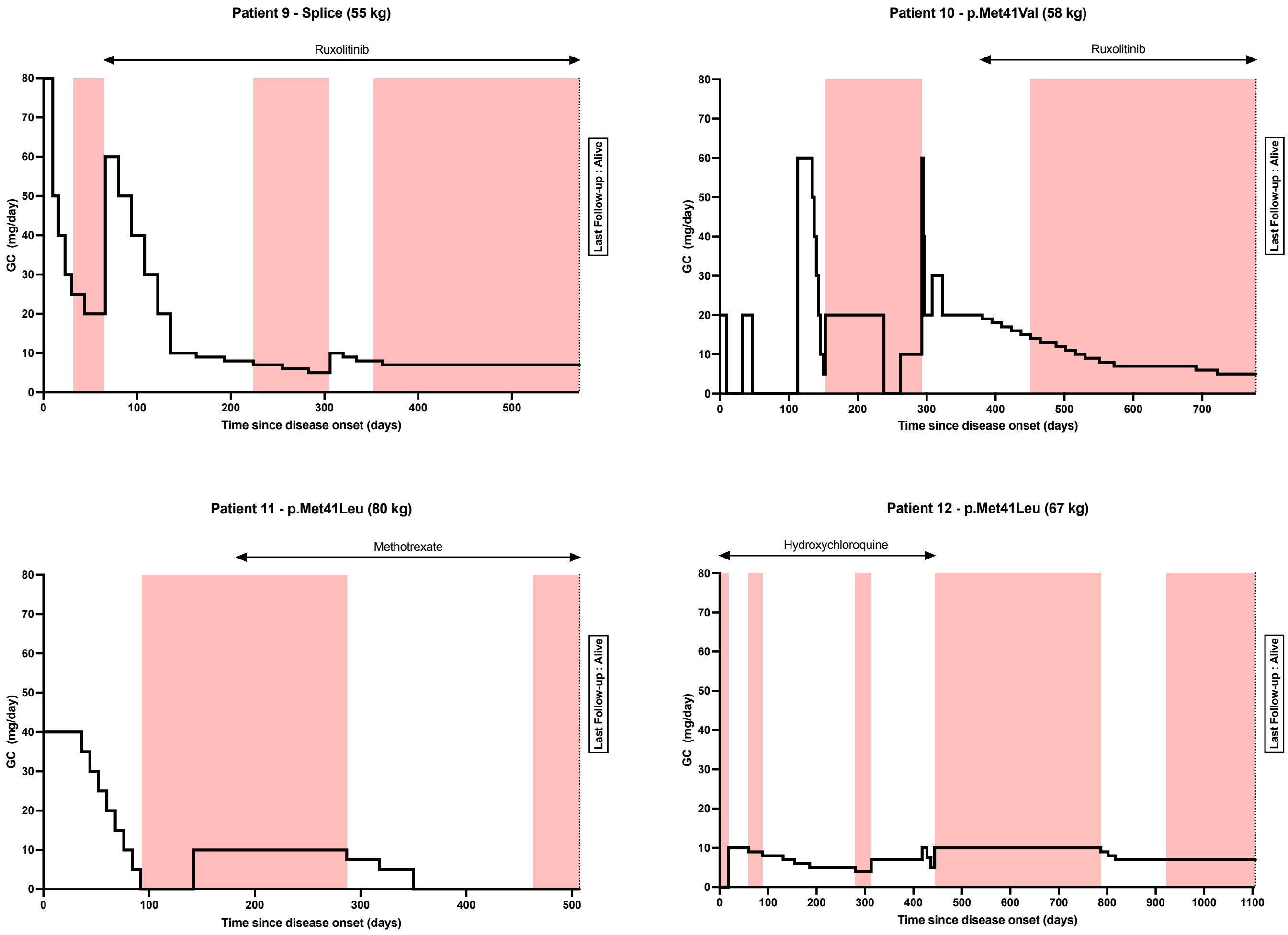

Patient 9 - Splice (55 kg)
Ruxolitinib
GC (mg/day)
Time since disease onset (days)
Last Follow-up : Alive
Patient 10 - p.Met41Val (58 kg)
Ruxolitinib
GC (mg/day)
Time since disease onset (days)
Last Follow-up : Alive
Patient 11 - p.Met41Leu (80 kg)
Methotrexate
GC (mg/day)
Time since disease onset (days)
Last Follow-up : Alive
Patient 12 - p.Met41Leu (67 kg)
Hydroxychloroquine
GC (mg/day)
Time since disease onset (days)
Last Follow-up : Alive
