# Supplementary figures and images for: Exploring glucocorticoid dose–response patterns in VEXAS syndrome: a pilot retrospective study
Source: Rheumatol Int. 2026 May 19;46(6):88. doi: 10.1007/s00296-026-06130-3 (PMC13186862; doi:10.1007/s00296-026-06130-3)

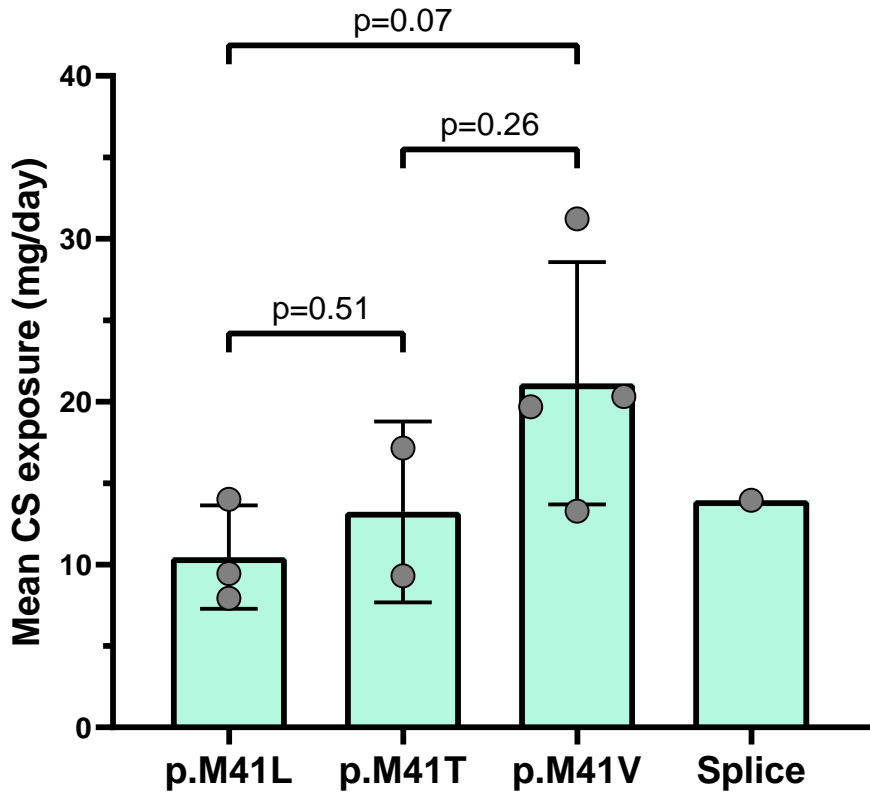

Supplement: Supplementary file 3 — Supplementary file3 (PDF 5 KB) Supplementary Data S3. Corticosteroid exposure according to UBA1 variant [file 296_2026_6130_MOESM3_ESM.pdf]
