# Supplementary material for: Exploring glucocorticoid dose–response patterns in VEXAS syndrome: a pilot retrospective study
Source: Rheumatol Int. 2026 May 19;46(6):88. doi: 10.1007/s00296-026-06130-3 (PMC13186862; doi:10.1007/s00296-026-06130-3)
Supplement: Supplementary file 4 — Supplementary file4 (PDF 191 KB) Supplementary Data S4. Multivariable logistic regression assessing the association between glucocorticoid dose and overall disease activity. Notes: Results are presented as adjusted odds ratios (aORs) with 95% confidence intervals. The model included glucocorticoid dose categories and concomitant therapies as independent variables. Treatments were analyzed individually: azacitidine, ruxolitinib, tocilizumab, anakinra or canakinumab, infliximab or etanercept, tofacitinib, methotrexate, cyclophosphamide, other, or none. Reference category was >40 mg/day for GC dose. GC: glucocorticoids [file 296_2026_6130_MOESM4_ESM.pdf]

<u>Supplementary Data S4</u>. Multivariable logistic regression assessing the association between glucocorticoid dose and overall disease activity

	p-value	aOR
GC >40 mg/day	Ref.	-
GC 30-39 mg/day	0.51	-
GC 20-29 mg/day	0.26	-
GC 15-19 mg/day	0.51	-
GC 10-14 mg/day	**<0.001**	13.8 [95% CI: 4.0-47.8]
GC 5-9 mg/day	**0.006**	6.2 [95% CI: 1.7-22.5]
GC <5 mg/day	**<0.001**	58.6 [95% CI: 14.7-233.6]

<u>Notes</u>: Results are presented as adjusted odds ratios (aORs) with 95% confidence intervals. The model included glucocorticoid dose categories and concomitant therapies as independent variables. Treatments were analyzed individually: azacitidine, ruxolitinib, tocilizumab, anakinra or canakinumab, infliximab or etanercept, tofacitinib, methotrexate, cyclophosphamide, other, or none. Reference category was >40 mg/day for GC dose. GC: glucocorticoids.
