# Supplementary material for: Exploring glucocorticoid dose–response patterns in VEXAS syndrome: a pilot retrospective study
Source: Rheumatol Int. 2026 May 19;46(6):88. doi: 10.1007/s00296-026-06130-3 (PMC13186862; doi:10.1007/s00296-026-06130-3)
Supplement: Supplementary file 5 — Supplementary file5 (PDF 206 KB) Supplementary Data S5. Multivariable logistic regression assessing the association between glucocorticoid dose and disease activity by clinical manifestation. Notes: Each column corresponds to a separate logistic regression model for a specific symptom. All models included glucocorticoid dose categories and concomitant therapies as independent variables. Treatments were analyzed individually: azacitidine, ruxolitinib, tocilizumab, anakinra or canakinumab, infliximab or etanercept, tofacitinib, methotrexate, cyclophosphamide, other, or none. Reference categories were <5 mg/day for glucocorticoid dose and no concomitant treatment. Constitutional symptoms include night sweats and other nonspecific systemic signs (e.g., fatigue, weight loss). GC: Glucocorticoids [file 296_2026_6130_MOESM5_ESM.pdf]

Supplementary Data S5. Multivariable logistic regression assessing the association between glucocorticoid dose and disease activity by clinical manifestation

	Fever	Constitutional symptoms	Chondritis	Joint	Ocular	Skin	Lung	Requiring hospitalization
GC >40 mg/day	**<0.001**	0.99	0.99	**0.007**	0.99	**<0.001**	**0.009**	**<0.001**
GC 30-39 mg/day	**0.006**	0.28	0.99	0.99	0.99	**<0.001**	**0.03**	**0.003**
GC 20-29 mg/day	**<0.001**	0.12	0.07	0.99	**0.04**	**<0.001**	**0.009**	**<0.001**
GC 15-19 mg/day	**0.005**	0.59	0.99	0.99	0.99	**<0.001**	**0.05**	**0.004**
GC 10-14 mg/day	0.128	0.78	**0.04**	**0.02**	0.24	**0.02**	0.13	**0.01**
GC 5-9 mg/day	**0.004**	0.36	0.35	**0.02**	0.39	**0.002**	0.26	**<0.001**
GC <5 mg/day	Ref.	Ref.	Ref.	Ref.	Ref.	Ref.	Ref.	Ref.

Notes: Each column corresponds to a separate logistic regression model for a specific symptom. All models included glucocorticoid dose categories and concomitant therapies as independent variables. Treatments were analyzed individually: azacitidine, ruxolitinib, tocilizumab, anakinra or canakinumab, infliximab or etanercept, tofacitinib, methotrexate, cyclophosphamide, other, or none. Reference categories were <5 mg/day for glucocorticoid dose and no concomitant treatment. Constitutional symptoms includes night sweats and other nonspecific systemic signs (e.g., fatigue, weight loss). GC: Glucocorticoids.
