# Supplementary material for: Exploring glucocorticoid dose–response patterns in VEXAS syndrome: a pilot retrospective study
Source: Rheumatol Int. 2026 May 19;46(6):88. doi: 10.1007/s00296-026-06130-3 (PMC13186862; doi:10.1007/s00296-026-06130-3)
Supplement: Supplementary file 6 — Supplementary file6 (PDF 181 KB) [file 296_2026_6130_MOESM6_ESM.pdf]

Statistical analyses

We conducted two complementary analyses:

- Disease activity analysis, in which all treatment-line periods were considered as either "active" or "inactive". For periods that included a flare, the time before flare onset was considered inactive, and the time after onset was considered active. This approach aimed to identify which treatments—and particularly which corticosteroid doses—were associated with disease control.

- Flare analysis, which focused exclusively on treatment-line periods classified as flares, i.e. periods that began in an inactive state and transitioned to active, compared with inactive periods. This analysis aimed to determine which treatment conditions—including corticosteroid doses—were associated with disease relapse.

Multivariable analyses were performed using logistic regression models to assess the association between GC dose and disease activity (overall and by clinical domain), adjusted for concomitant treatments analysed individually (azacytidine, ruxolitinib, tocilizumab, anakinra or canakinumab, infliximab or etanercept, tofacitinib, methotrexate, cyclophosphamide, other treatment, or no additional treatment). Adjusted odds ratios (aORs) and 95% confidence intervals (95% CIs) were calculated to quantify associations.

As a sensitivity analysis, the main model assessing the association between GC dose and disease activity was repeated using a mixed-effect logistic regression, including patient ID as a random intercept, to account for intra-patient clustering, and adjusting for concomitant therapies.

GC dose was considered either as a continuous variable or as a categorical variable (<5, 5–9, 10–14, 15–19, 20–29, 30–39, ≥40 mg/day). The reference category was selected according to the analytical objective: either <5 mg/day without concomitant immunosuppressive therapy

(to assess the protective effect of increasing GC exposure), or ≥40 mg/day (to define a maximal GC treatment and evaluate the relative risk of disease activity or flare during steroid tapering).

Maximally selected rank statistics based on standardized log-rank tests were applied to determine the GC dose threshold that best discriminated between flare and non-flare periods.

Time-to-event analyses were performed using Kaplan–Meier survival curves and compared with log-rank tests. Hazard ratios (HRs) and 95% CIs were estimated using Cox proportional hazards models, adjusting for treatment regimen and GC dose. Treatments were analyzed either individually or grouped into three categories based on their documented level of efficacy: Group 2 (azacytidine, ruxolitinib or tocilizumab, which demonstrated higher efficacy in VEXAS), Group 1 (other immunosuppressive agents), and no additional treatment.

Quantitative data are presented as mean ± SD, and categorical data as counts and percentages. A two-sided type I error rate of 5% was applied. All analyses were performed using GraphPad Prism v8.0.2 and Jamovi v2.3.9.
